# Fabrication and evaluation of vitamin doped Zno/AgNPs nanocomposite based wheat gluten films: a promising findings for burn wound treatment

**DOI:** 10.1038/s41598-023-43413-2

**Published:** 2023-09-26

**Authors:** Anila Sajjad, Hussain Ali, Muhammad Zia

**Affiliations:** 1 Department of Biotechnology, Quaid-i-Azam University, Islamabad, 45320 Pakistan; 2 Veterinary Farms Management Sub-Division, National Institute of Health, Islamabad, Pakistan

**Keywords:** Biotechnology, Health care, Materials science

## Abstract

Burn wound treatment remains a significant issue in wound care management especially when multidrug resistant bacterial infection and accumulation are present. Delayed wound healing is mostly due to ineffectiveness of commercially available wound dressings that protects the wound but less efficient in healing perspective. Therefore, nano-based wound dressing might be efficient solution for wound healing management. The present study reports the fabrication and evaluation of zinc oxide (ZnO) or silver nanoparticles (Ag NPs) capped with vitamin A or E nanocomposite that were incorporated in wheat gluten (WG) films. The chemical structure, phase purity, and morphological features confirmed the successful coating of NPs by vitamins A and E and their interaction with WG during film casting. The maximum swelling response was observed by NPs vitamin composite WG films than control films while slow release of vitamins and NPs from films was observed up to 24 h. WG films either carrying ZnO or Ag NPs, and vitamin A or E demonstrated significant antioxidant and antibacterial potential. The NPs-vitamin composite loaded WG films showed wound contraction within 14 days during in vivo burn wound healing experiments on mice model. The rates of wound healing, re-epithelialization, collagen deposition with fibroblast regeneration, adipocytes, and hair follicle development were observed through visual and histopathological examination. The study reveals that vitamin A or E doped ZnO or Ag NPs fabricated in WG can be efficiently used against burn wounds due to their physiochemical and biological properties. Furthermore the biocompatible nature and biodegradable potential make the films more prone to mankind maneuver for initial protection and healing remedy.

## Introduction

A wound is defined as a disruption in the integrity or dysfunction of skin tissue caused by trauma or burn that may significantly affect the patient’s life quality. However well-timed protection and a rapid healing process are critical for preventing prolonged damage^1^. Despite various advancements, wound management remains a significant clinical and economic issue. According to the recent report, the global market for wound treatment products is predicted to grow from US$ 19.83 billion in 2020 to US$ 30 billion by 2027^2^. Although these dressing products, are less structurally stable and efficient against multidrug-resistant (MDR) pathogens, affecting the patients' overall health. Currently, one of the key factors influencing wound healing is the ineffectiveness of commercially available wound dressing materials^3^. To meet clinical standards, several desirable properties should be addressed for the transdermal modalities such as economical, user-friendly, appropriate adherence, mechanical strength, superabsorbent, biocompatible, and biodegradable^4–6^. The major goal of transdermal delivery systems is to develop skin formulations with optimum flow through the skin layers by which bioactive agents can penetrate through the skin by passive diffusion. Therefore, the formulations should only be loaded with agents that have moderate lipophilicity and possess anti-infective, anti-inflammatory, super-absorbency, and wound-healing properties^7^. For these reasons, wound dressings such as foams, hydrogels, nanofibers, and nanofilms have been developed for the transdermal delivery of novel bioactive agents over conventional modalities^8,9^.

The majority of wound dressings used today are made from a combination of bio-polymers and synthetic polymers to enhance the structural and functional features of the formulation. Wheat gluten (WG) protein is one of the most significant biopolymers and is divided into two protein fractions based on solubility: gliadins and glutenins. Wheat gluten is treated in various chemical and biological ways to increase its solubility, leading to partial hydrolysis^10^. Wheat gluten can be a promising choice because of its easy availability, polymerization process, biodegradability, and film-forming properties^11,12^. Recently, wheat gluten films have been functioning as a drug delivery vehicle for the progressive release of antioxidants and antibacterial substances to enhance the structural, chemical, and functional properties of films^13^.

The applications of inorganic nanoparticles (NPs) with antioxidative and antibacterial properties have gained their clinical efficacy^14^. ZnO is a biocompatible metal nanoparticle that has antibacterial, anti-infection, immune regulation, and tissue regeneration properties^15,16^. Additionally, ZnO is less toxic and serves as a source of Zn, that is required by 300 catalytic enzymes that influence cell differentiation and proliferation as well as wound healing^17^. Likewise, Ag NPs have gained a new therapeutic potential to be employed in wound dressings owing to their safety, reliability, low toxicity, potency, and resistance to various bacteria^18^. The unique physicochemical properties of these NPs are suitable for enhanced encapsulation, active and passive targeted delivery, and regulated and sustained drug release^19,20^. Therefore, various nano-based dressinghave been fabricated by the integration of NPs with different biocompatible polymers, like chitosan, polylactic acid (PLA), polyvinyl pyrrolidone (PVP), and polyvinyl alcohol (PVA), for efficient wound healing applications^18,19,21^.

Compounds that inhibit oxidation in biological systems by minimizing the incidence of oxidizing processes are recognized as antioxidants^22^. Vitamins are well known for their antioxidant properties and ability to accelerate wound healing^23^. Vitamin A palmitate (VA) is a lipid-soluble compound used to treat skin ailments. It is more stable and can be turned into vitamin A (retinol) under physiological conditions^23^. Vitamin E (Alpha-tocopheryl acetate, VE), another lipid-soluble vitamin, protects cellular membranes and unsaturated fatty acids from reactive oxygen species (ROS) by activating multiple signal transduction pathways and is widely acknowledged for its antioxidant properties^1,24^. VA and VE boost collagen synthesis in the wound healing zone^23,25,26^. Many wound healing dressings have been designed with the embedment of metal/metal oxide NPs and other bioactive agents, including nano ZnO^27^, ZnO-chitosan^28^, citric acid coated ZnO nanofilms^29^, Ag nano-scaffolds^18^, chitosan-sericin-Ag nanocomposite^21^, gelatin-based nanofibers loaded with vitamin A and E^23^, chitosan alginate hydrogel containing vitamin E^1^, retinoic acid loaded lipid nanoparticles^30^, cellulose acetate nanofibers loaded with vitamins (A and E)^31^, etc. However, several of these wound dressings lack the structural integrity, and flexibility required to cover the wound, limited biological properties, may inhibit cell proliferation, are non-biodegradability, and high cost^32–34^. Therefore fabrication of a suitable biologically active wound dressing for treating infected wounds is indispensable and nanocomposites might be an effective technique^35^.

In this study, film casting technique was used to incorporate ZnO NPs and Ag NPs conjugated with vitamin A and E into wheat gluten films. Based on the in vitro antioxidant and antibacterial activity, swelling capacity, and sustained release of NPs and vitamins of fabricated films, in vivo studies were conducted to validate the films' ability to heal burnt skin in mice model.

## Experimental

### Materials

Whole-grain wheat flour was used for the extraction of wheat gluten (WG). Zinc acetate dihydrate (Zn (CH_3_COO)_2_·2H_2_O), Silver nitrate (AgNO_3)_, sodium borohydride, sodium hydroxide (NaOH), sodium dodecyl sulfate (SDS), 2, 2-diphenyl-1-picrylhydrazyl (DPPH) were of analytical grade purchased from Sigma Aldrich, USA. Glycerol (CH_2_OHCHOHCH_2_0H; Molecular Weight 92.09 g/mol) and gellan gum powder were supplied by Merck, pharma. Poly (vinyl pyrrolidone) (PVP) average molecular Wt. 130 KD, and poly(vinyl alcohol) (PVA) granules Molecular wt. 125 KD were purchased from Sigma Aldrich. Vitamin A palmitate and vitamin E (Alpha tocopheryl acetate) were purchased from BASF, Hong Kong.

### Synthesis of ZnO NPs and Ag NPs

ZnO NPs were synthesized by dropwise mixing 50 ml zinc acetate (1 mM) with 50 ml NaOH (2 M) solution under continuous stirring for 3 h. The white precipitate was separated by centrifugation at 2000*g* for 20 min and washed thrice with distilled water. The NPs were oven dried at 40 °C and stored^36^.

Ag NPs were synthesized using the co-precipitation method following the protocol by Song et al.^37^. In brief, 50 ml AgNO_3_ (1 mM) was dropwise added to 50 ml of NaBH_4_ and SDS (1% each) solution. The mixture was stirred at 1000 rpm for 1 h at room temperature in the dark. The grayish-black precipitate was centrifuged at 10,000 g for 10 min. The precipitate was washed thrice with distilled water and dried in a vacuum oven at 40 °C.

### Wheat gluten extraction and solubility

The standard procedure was followed to extract WG from whole wheat flour^38^. Briefly, the dough was prepared using 20 g wheat flour. The dough was wrapped in a cotton cloth and thoroughly rinsed under running tap water to remove all starch. Wheat gluten WG, the resultant rubbery substance, was dried at 40 °C in an incubator. The dried pellet was ground into powder by the electric grinder. To hydrolyze wheat gluten, 1.5 g WG powder was suspended in 100 ml of 1 mM NaOH solution. The suspension was agitated at room temperature for 30 min at 300 rpm and heated in a water bath at 50 °C for 90 min thereafter.

### Surface modification of NPs by vitamin coating

For preparing the NPs vitamin composites, 1 g/100 ml vitamins A and E were separately dissolved in Ethanol + Distilled water (1:500). In vitamin E solution Tween 20 (0.01%) was also added. The solutions were stirred until the vitamins were completely dissolved. In each vitamin solution, Ag NPs and ZnNPs (at 1% each separatl) were added to formulate NPs vitamin nanocomposite denoted as Zn/A, Zn/E, Ag/A, and Ag/E. The composite solutions were further stirred for 36–48 h at room temperature for successful coating. After stirring, the nanocomposite was purified by centrifugation and washed twice with distilled water. The resultant pellet was dried in the incubator at 37 °C and kept in airtight glass vials.

### Casting of WG films

For the film casting, the traditional solvent evaporation method was used^20^. The reference WG films were casted by first dissolving 0.5% each of PVP, and PVA in 15 ml distilled water, then adding gellan gum (0.3%) and glycerol (0.2%) to each polymeric mixture. After 2 h stirring, 25 ml of hydrolyzed WG (1.25%) was added to each solution, yielding a total volume of 40 ml. The mixture was stirred for 4 h at room temperature. The final gel-like solution was then transferred into Petri plates, dried at 40 °C for 24 h, and gently peeled off thereafter. The same procedure was used for the nanocomposite WG films. For that 0.05% NPs were added to the WG solution followed by sonication for an hour in a water bath at room temperature. For the preparation of vitamin-loaded nanocomposite films, 0.05% of each NPs-vitamin composite (Zn/A, Zn/E, Ag/A, and Ag/E) was added to the WG solution separately and stirred for an hour. The NPs vitamin WG composite solution was added to the PVP, and PVA solutions to form WG/P/Zn/A, WG/P/Zn/E, WG/A/Ag/A, and WG/A/Ag/E, respectively. The solutions were further stirred for 6 h. After stirring time, the obtained mixtures were poured into the petri plates, dried at 40 °C for 24 h, and gently peeled off. The films were stored at 4 °C until characterizations were performed. Table 1 represents the all prepared films and their nomenclature.

**Table 1:** Variously prepared Wheat gluten films and their codes are used from now in the article.

Wheat gluten films	Codes
Wheat gluten-PVP	WG/P
Wheat gluten-PVA	WG/A
Wheat gluten-PVP with 0.05% ZnNPs	WG/P/Zn
Wheat gluten-PVA with 0.05% Ag NPs	WG/A/Ag
Wheat gluten-PVP with 0.05% ZnNPs + vitamin A	WG/P/Zn/A
Wheat gluten-PVP with 0.05% ZnNPs + vitamin E	WG/P/Zn/E
Wheat gluten-PVA with 0.05% Ag NPs + vitamin A	WG/A/Ag/A
Wheat gluten-PVA with 0.05% Ag NPs + vitamin E	WG/A/Ag/E

### Characterization of nanoparticles and WG films

The Perkin Elmer spectrum was used for FTIR spectral analysis in diffuse reflection mode with a resolution of 4 cm^−1^, and the spectrum was collected in the range of 4000–400 cm^−1^. The images from the Field Emission-Scanning Electron Microscopy (FE-SEM) were taken at 100KX magnification using a 20 kV electron beam TESCAN MIRA3 LMH Schottky (USA). Before SEM analysis, a small section of the films was cut, put on a sample holder, and coated with gold to determine the surface features of the films. X-ray diffraction (XRD) analysis was used to determine the size and crystal phases of nanoparticles. The XRD patterns of nano-vitamin loaded WG films were obtained on a powder EMPYREAN diffractometer operating at 45 kV and 40 mA with Cu Ka radiation λ = 0.15418 nm over a 10° to 80° of 2θ range.

### Physical features analysis

The transparency or opacity of the films was examined visually. The thickness of films was measured at both, central and peripheral areas using a calibrated digital micrometer. By weighing each film, the average weight difference was noted. Folding efficiency was determined by folding a piece of film several times at 180° angle.

The swelling test, and the swelling response (%) was evaluated using a gravimetric method^39^. Each film sample (2 × 2 cm^2^) was weighed before and after immersion in 20 ml of phosphate buffer (pH 7). During the 12 h of immersion at 37 °C, the film samples were periodically removed and placed on filter paper to soak extra water before the wet weight (Ww) was measured. The weight variations were recorded in triplicate and % swelling was calculated by using the equation.

$${\text{Swelling}}\,{\text{response }}\left( \% \right) \, = {\text{ Ww }}{-}{\text{ Wd }}/{\text{ Wd }} \times {1}00$$

### In vitro release of NPs/ions vitamins and release kinetics

The immersion method was employed to investigate the release of NPs/ions and vitamins (A and E) from nanocomposite WG films. In each flask, a 50 mg film sample was placed in 200 ml of phosphate buffer solution (PBS) of pH 7. The flasks were stirred at 37 °C for 24 h at 100 rpm using a magnetic stirrer. At regular intervals, 2 ml was collected from each flask and absorbance was measured at 450 nm for Ag NPs^20^, and 340 nm for ZnNPs^20,40^. The flasks were refilled with the same amount of PBS (2 ml) at each sampling to keep the fluid level constant. For vitamin release estimation, VE and VA were then evaluated at 285 and 330 nm^23,31^, respectively using a UV spectrophotometer, against a preset calibration curve for each vitamin. The data were calculated to find the percent of vitamins released from each sample. The experiments were conducted in triplicate, and the findings were presented as average values.

$${\text{Release }}\left( \% \right) \, = {\text{ amount}}\,{\text{of}}\,{\text{drug}}\,{\text{released}}\,{\text{in}}\,{\text{medium}}/{\text{amount}}\,{\text{of}}\,{\text{drug}}\,{\text{loaded}}\,{\text{into}}\,{\text{the}}\,{\text{film }} \times {1}00$$

To understand the release mechanism, the release data were fitted to the Korsmeyer–Peppas model equation^21^. Mt/M∞ = Ktn Where Mt/M∞ is the fraction of NPs/ions or vitamins released at time t, K is the rate constant and the exponent “n” represents the NPs/ions and vitamins transport mechanism and is used to evaluate the mechanism of diffusion.

### In vitro Biological potential of nanocomposite WG films

The disc diffusion approach was followed to examine the antibacterial activity of films against *Pseudomonas aeruginosa* (ATCC-15442), *Escherichia coli* (ATCC-25922), *Klebsiella pneumoniae* (ATCC-1705), *Staphylococcus aureus* (ATCC-6538), *Methicillin-resistant Staphylococcus aureus (MRSA*, ATCC-43300), and *Staphylococcus epidermidis* (ATCC-12228). The 6 mm of small discs were made from the films and implanted on agar plates containing bacteria. The agar plates were incubated at 37 °C for 24 h, and the zone of inhibition (ZOI) was measured. The micro broth dilution (MBD) method was used to test the films' potential to prevent bacterial growth. In a 96-well plate, a 6 mm disc from each film was placed into each well, followed by the addition of 200 µl of bacterial culture of 0.1 optical density (OD), and incubated for 24–36 h at 37 °C^20^. The OD value was recorded at 630 nm and computed to find out the bacterial growth inhibition (%) by the following equation.

$${\text{Inhibition }}\left( \% \right) \, = {\text{ Abs}}\,{\text{control }}{-}{\text{ Abs}}\,{\text{sample}}/{\text{Abs}}\,{\text{control }} \times {1}00$$

The radical scavenging potential of WG films was tested using 2,2-diphenyl-1-picrylhydrazyl (DPPH) reagent and 2,2′-azino-bis ethylbenzthiazoline-6-sulfonic acid (ABTS^+^) radical cation following the protocol reported by Sajjad et al.^41^.

### In vivo evaluation of burn wound healing

**Figure 1:**
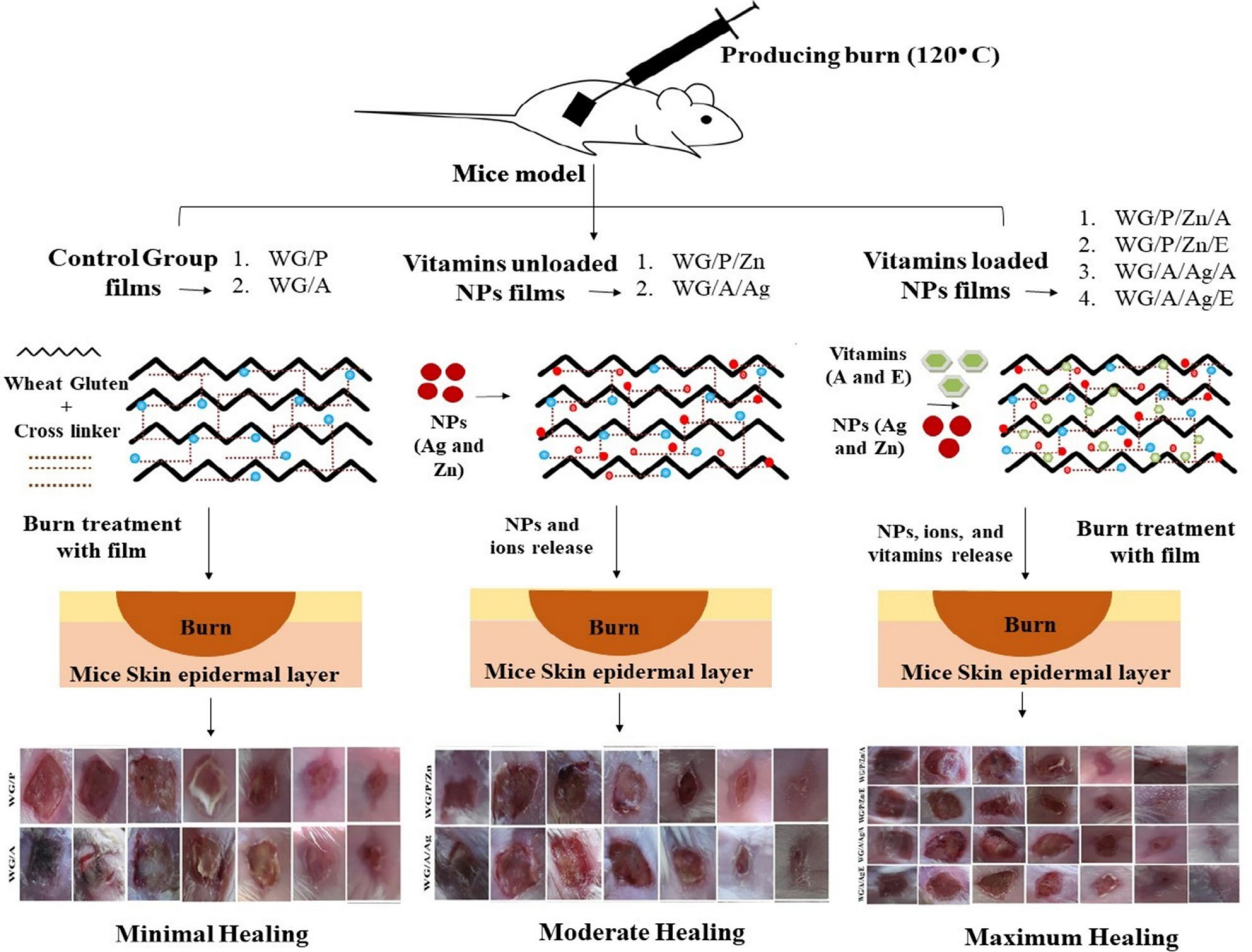
Pictorial presentation of mice model burn healing experiment.

The ethical approval for the present study was granted by the Institutional Ethics Committee for Animal Care and Experimentation (IECACE; F.1-5/ERC/2022) established by the National Institute of Health (NIH) Islamabad regarding general care, handling, and the use of laboratory animals. The guidelines for animal studies follow the regulations defined by the European Union (www.eara.eu) and ARRIVE (www.arriveguidlines.org). The mice animals were categorized into nine groups each group containing six mice and all were female (30–35 g average weight). The number of mice in each group were selected based on the literature^42^. Before the burn wound was made, the mice were anesthetized with an intraperitoneal injection of ketamine (75 mg/kg) + Xylazine (5 mg/kg) based on body weight^3^. Hair was meticulously shaved off the dorsal surface of the mice, and a single burn wound (8 mm^2^) was created on the dorsum of each mouse by placing a square-shaped metallic block (heated to 120 °C) for 10 s without applying any force. The wheat gluten films were sliced into 10 mm^2^ pieces. Group I was treated as the negative control (no treatment). Groups II and III were treated with WG/P and WG/A films. Groups IV and V were treated with, WG/P/Zn and WG/A/Ag films. Groups VI, VII, VIII, and IX were treated with WG/P/Zn/A, WG/P/Zn/E, WG/A/Ag/A, and WG/A/Ag/E, respectively. All films were topically applied to the burn wound models as wound-healing dressings. An adhesive bandage was used to fix the dressings to the injured site. The treatments were given to each animal in the treated groups on the day of burn induction (day 0), and the treatments were reapplied every 24 h as per the 14-day research plan. The wound area was sterilized with 70% ethyl alcohol and 0.9% saline solution before applying the dressing. The physical characteristics of the animals, such as their routine behavior, walking, and standing on their hind legs without showing any signs of pain, as well as the lesion-induced skin changes, such as inflammation, redness, swelling, blistering, and bleeding, were closely observed throughout the experiment^21^. The wound healing criterion was evaluated as percent wound closure and histological analysis of skin tissues taken after the animals were sacrificed on the 14th day of the study. The methodology, experimental groups, and outcomes are illustrated in Fig. 1.

The rate of wound contraction was assessed on the pre-specified days, i.e. the 3rd, 5th, 7th, 9th, 11th, and 14th by measuring the wound area with a vernier caliper^18^. The wound closure, represented as a percentage (%) of wound contraction, was computed as follows:

$$\% \,{\text{wound}}\,{\text{contraction}}\, = \,\left( {{\text{wound}}\,{\text{area}}\,{\text{on}}\,{\text{the}}\,{\text{initial}}\,{\text{day}} - {\text{wound}}\,{\text{area}}\,{\text{on}}\,{\text{a}}\,{\text{specific}}\,{\text{day}}} \right)/{\text{wound}}\,{\text{area}}\,{\text{on}}\,{\text{initial}}\,{\text{day}} \times {1}00.$$

### Histopathological analysis

On the 14th day, three skin tissue samples from the treated area of each mouse were excised following instantaneous sacrifice, rinsed with distilled water, and fixed in 10% formalin solution for preservation of tissue and histological examination. The epithelial tissues were stained with hematoxylin and eosin (H & E). The prepared glass slides were examined under light microscope to analyze tissue architecture and remodeling, as well as to observe the state of inflammation, collagen reorganization, hair follicles, fibroblast, and epidermal regeneration.

### Statistical analysis

All experiments were carried out in triplicate, and the findings are shown as mean with standard deviation. Moreover, the results were statistically analyzed using the least significant difference (LSD) with a probability of 0.05 percent.

### Ethical approval

The ethical approval for the present study was granted by the Institutional Ethics Committee for Animal Care and Experimentation (IECACE; F.1-5/ERC/2022) established by the National Institute of Health (NIH) Islamabad regarding general care, handling, and the use of laboratory animals. The guidelines for animal studies follow the regulations defined by the European Union (www.eara.eu) and ARRIVE (www.arriveguidlines.org).

### Consent to participate

Manuscript does not contain human-related data therefore consent to participate is not required.

## Results and discussion

### FTIR analysis of WG films

**Figure 2:**
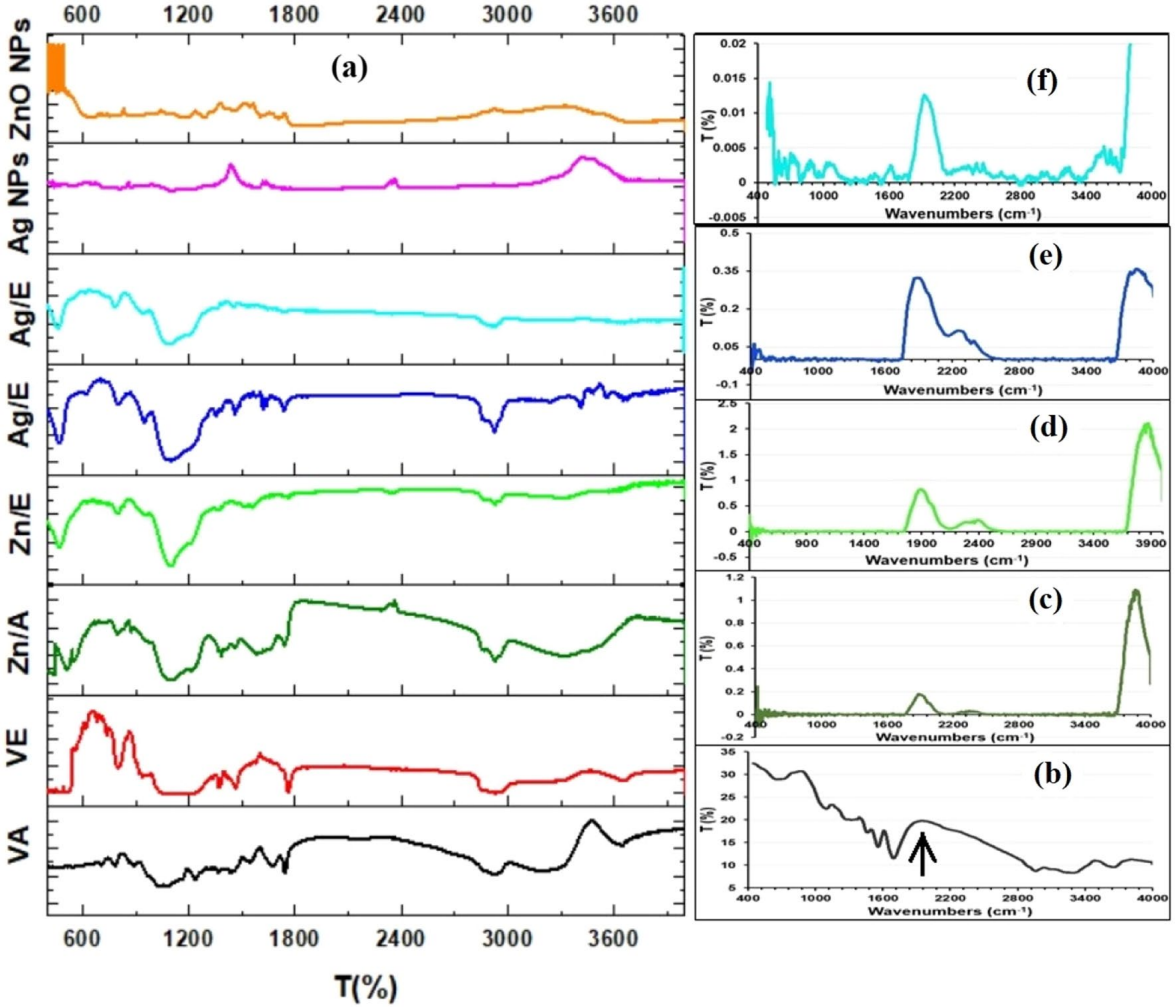
FTIR spectra of pure vitamin A, vitamin E, vitamin-coated nanocomposites (Zn/A, Zn/E, Ag/A, and Ag/E), ZnO NPs, Ag NPs, pure wheat gluten powder, and NPs-vitamin loaded WG films (WG/P/Zn/A, WG/P/Zn/E, WG/A/Ag/A, and WG/A/Ag/E).

The spectrum of the VA (Fig. 2) shows the characteristic bands at 2925 cm^−1^ that represent aliphatic C–H stretching, bands at 1749 and 1675 cm^−1^ correspond to O–C=O esters bond and carbonyl (C=O) from carboxylic acids; the peaks in the region of 1540 and 1444 cm^−1^ are characteristic of the ethylenic interactions (C=C); the stretching between 1236–1076 cm^−1^ is attributed to the C–O bond of carboxylic acids and the broad band between 3200 and 3300 cm^−1^ relates to O–H stretching vibrations^30^. FTIR spectrum of both vitamin A-coated ZnNPs (Zn/A) and Ag NPs (Ag/A) showed characteristic peaks of VA and NPs. Zn/A showed characteristic VA peaks by C–H stretching vibration at 2925 cm^−1^, a stretch of ester bond (O–C=O) at 1741 cm^−1^, and C–O formation for carboxylic acid at 1106 cm^−1^ (Fig. 2) For ZnNPs in Zn/A composite, characteristic M–O peaks were present at 500 cm^−1^ and 800 cm^−1^ due to the formation of tetrahedral coordination of Zn^43^. However, the band at around 3200–3500 cm^−1^ is assigned to the O–H stretching vibrations in Zn/A nanocomposite. The characteristic VA peaks in Ag/A nanocomposite were observed at 2929 cm^−1^ representing the aliphatic C–H stretching, esteric bond formation at 1739 cm^−1^, and C=O carboxylic bond formation at 1112 cm^−1^. The characteristic peaks for Ag NPs in Ag/A nanocomposite spectrum were found in the region of 470–800 cm^−1^.

The VE spectrum exhibited characteristics bands at wavelengths 3350–3150 cm^−1^ for terminal –OH group, 2954 cm^−1^ for C–H stretching vibrations of alkanes, 1770 cm^−1^ represent C=O stretching of ester bond, 1467 and 1380 cm^−1^ for CH_3_ symmetric and asymmetric bending, broadband at 1214–1091 cm^−1^ is due to the C–O stretching of phenols, and 998 and 808 cm^−1^ for trans = CH_2_ stretching^44,45^. Vitamin E was successfully coated on ZnNPs and Ag NPs and it was confirmed by the presence of VE characteristics peaks in Zn/E and Ag/E nanocomposites spectrum (Fig. 2). The broad absorption band at 3400–3650 cm^−1^ is attributed to the terminal hydroxyl group of VE which demonstrates substantial numbers of hydrogen bonds in the chemical structure of Zn/E and Ag/E^35^.

The gluten spectrum represents the characteristics bands at 1683 cm^−1^, 1540 cm^−1^, and 1500 cm^−1^ corresponding to amide I (carbonyl stretching) and amide II and the intrinsic protein secondary structure^46^, respectively (Fig. 2). NPs vitamin loaded WG films exhibit prominent bands between 3700 and 4000 cm^−1^ that are assigned to the O–H group of glycerol^47^. The peaks at 2345 cm^−1^ (WG/P/Zn/E film) and 2260 cm^−1^ (WG/A/Ag/E film) show C–N stretching vibrations due to the presence of amine group in wheat gluten^47,48^. The new peaks observed in the NPs vitamin composite loaded WG films (Fig. 2) at around 1878–1913 cm^−1^ are assigned to the conformational changes of the protein secondary structure^49^ bonded on the NPs surface containing vitamin molecules. It also signifies the strong interactions (H-bonding) of the vitamins and NPs embedded within the WG matrix. The shifting of bands and changes in their intensities resulted in the formation of new peaks in the prepared films, confirming the complex network during film formation^13^. All the characteristic peaks of NPs were detectable in NPs vitamin composite loaded WG film spectra in the range of 400–500 cm^−1^.

### Characterization of nanoparticles

**Figure 3:**
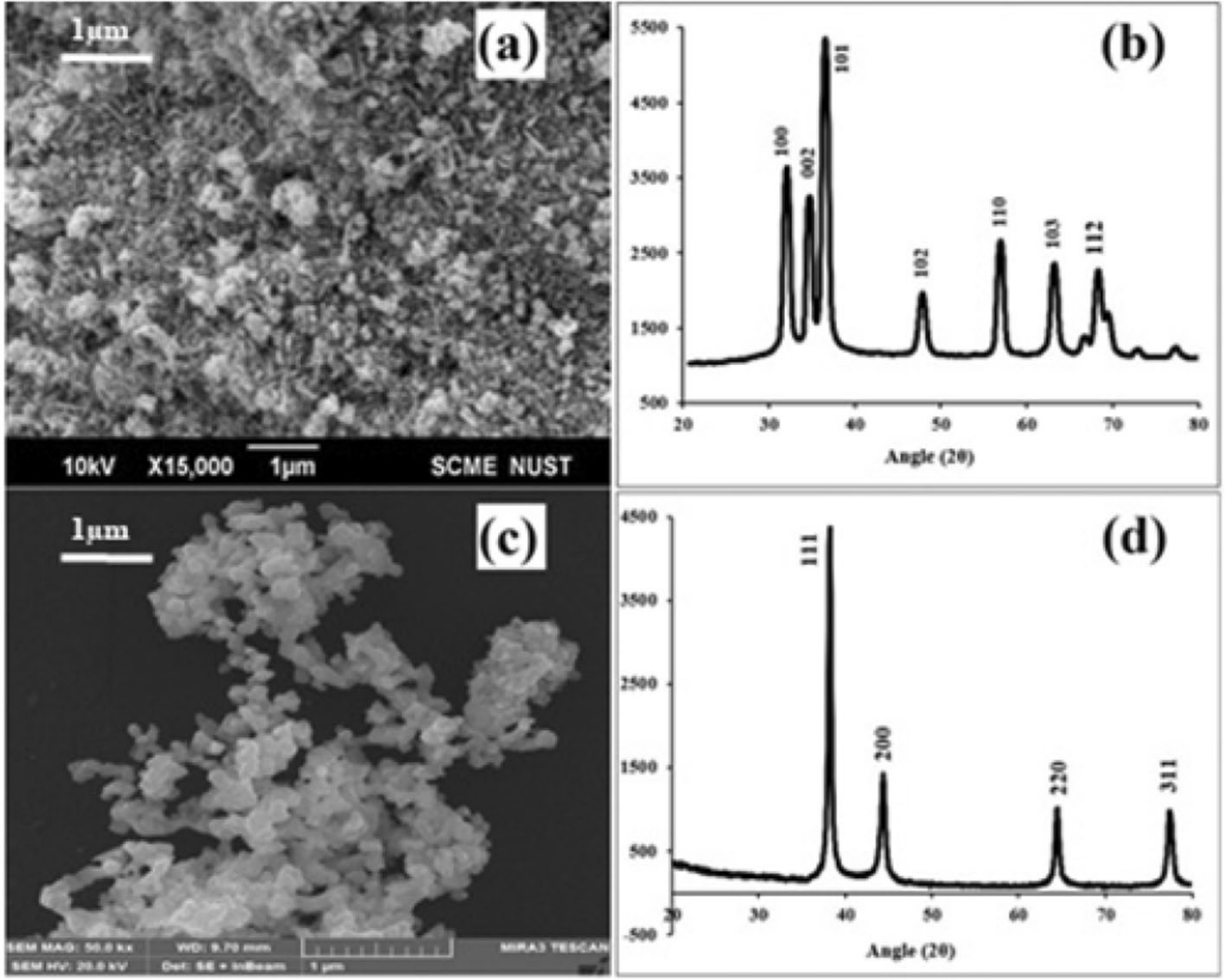
Morphology (SEM) and structural patterns (XRD) of ZnO and Ag NPs; (**a**) SEM image of ZnO NPs (**b**) XRD of ZnO NPs (**c**) SEM image of Ag NPs (**d**) XRD of Ag NPs.

SEM (Fig. 3a) demonstrate the spherical surface morphology of ZnO NPs with severe agglomeration. XRD patterns of Fig. 3b displays peaks at angles 2θ of 31.69°, 34.34°, 36.18°, 47.47°, 56.55°, 62.80°, and 67.91°, confirms the crystalline wurtzite (hexagonal) structure of ZnO NPs with an average crystallite size of 22 nm. Figure 3d shows the well-defined peaks at 2θ at 38.09°, 44.25°, 64.46°, and 77.42°, reveals the face-centered cubic structure of crystalline Ag NPs. The average particle size of Ag NPs was 16 nm and SEM images confirm the spherical shape of Ag NPs (Fig. 3c).

### Surface micro-morphology of WG films

**Figure 4:**
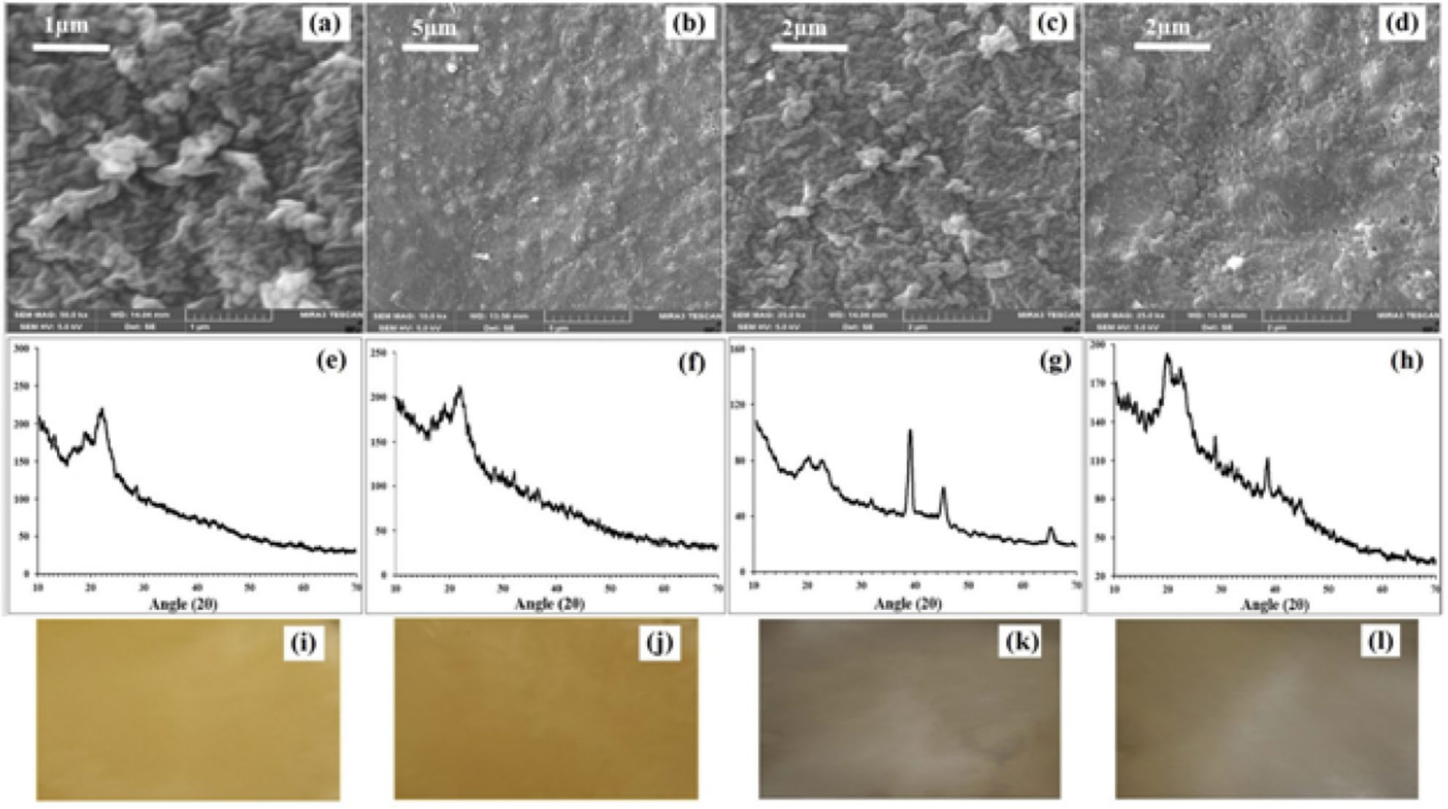
Characterization of NPs-vitamin loaded WG films. (**a**–**d**) Surface micro-morphology of WG/P/Zn/A, WG/P/Zn/E, WG/A/Ag/A, and WG/A/Ag/E. (**e**–**h**) Structural patterns of WG/P/Zn/A, WG/P/Zn/E, WG/A/Ag/A, and WG/A/Ag/E. (**i–l**) Prepared NPs-vitamin loaded WG films (WG/P/Zn/A, WG/P/Zn/E, WG/A/Ag/A, and WG/A/Ag/E).

Figure 4a–d depicts the surface characterization of gluten films fabricated with NPs and vitamins (A & E) using scanning electron microscopy. SEM images show the entrapment of NPs in the WG film. WG/P/Zn/A and WG/A/Ag/A films had rough and compact surface whereas WG/P/Zn/E and WG/A/Ag/E films were distinguished by rough surfaces with smaller pits and depressions. Notably, bumpy structures found on the film's surface made from the solution containing vitamin E (Fig. 4b,d) are thought to be the phase separation response of components during the film drying stage. It is also hypothesized that the vitamin E rich phase might be the reason for the development of these bumps^31^. The VE-NPs WG films (WG/P/Zn/E and WG/P/Ag/E) possessed homogenous pits/depressions inside the film structure, which might improve water absorption^1^. WG/P/Zn/A and WG/A/Ag/A films exhibited denser structures without pits which represent regular crosslinking between the WG, NPs, and vitamin A molecules (Fig. 4a,c). Taepaiboon et al.^31^ also found the compact structure of Retin-loaded cellulose acetate films. The films micrometric roughness may enable them to stick to the healing tissues, resulting in a greater contact surface and subsequently prolonging the duration that the films remain in touch with the lesion. This would enable the promotion of effective burn wound cicatrix. Furthermore, the presence of vitamins in nanocomposite WG films significantly modified the surface topography of the films based on vitamin characteristics. However, ZnNPs and Ag NPs aggregates were visible on the surface of NPs-vitamin-loaded WG films. The addition of spherical-shaped Ag NPs in WG/A film altered the film surface morphology with a smooth and compact structure, facilitating the slow release of ions from the film microstructure^20,50^. ZnO NPs in WG/P film were trapped on the film surface and certain gaps were visible in the cross-sectional image of the film which promoted the high ion release and maximum water uptake from the surroundings.

### Structural patterns of NPs-vitamin loaded WG films (XRD)

The structural patterns of NPs-vitamin loaded WG films were obtained by X-ray diffractogram (XRD) (Fig. 4e–h). The structural patterns of films confirm the reduction in crystallinity. The slow evaporation rate of the solvent during the solvent casting process may also cause the gluten molecules to crystallize in the casted films as they gradually diffuse to the crystal growth^51^. This is evident in the XRD graphs of NPs-vitamin loaded WG films (Fig. 4e–h), which displayed a sharp gluten peak at 2θ of 20°–22°. A gradual reduction in the crystallinity of ZnO NPs films was observed when vitamins A and E were incorporated into the film along with gluten. Similar results have been reported in our previous investigations, where lower intensities of Ag NPs and ZnO NPs were detected due to peak overlapping between wheat gluten and nanoparticles^20,40^.

Vitamin-loaded Zn films (WG/P/Zn/A, WG/P/Zn/E) diffraction patterns revealed ZnNPs peaks at an angle 2θ of 31°–67°, which are referenced as the hexagonal (wurtzite) structure of zinc oxide nanoparticles (Fig. 4e,f). These peaks correspond to the (100), (002), (101), (102), (110), (103), and (112) faces of ZnO NPs (JCPDS No. 36-1451). In the case of vitamins composite Ag NPs films (WG/A/Ag/A, WG/A/Ag/E), the diffraction pattern shows strong peaks at 38.09°, 44.25°, and 64.46°, respectively, corresponding to crystallographic planes (111), (200), and (220) (JCPDS No. 034-0783). This reflects that Ag NPs with face-centered cubic structures are crystalline and preserved during the film formation (Fig. 4g,h). However, Ag-vitamin WG films exhibited well-defined peak patterns as compared to Zn-vitamin WG films. The intensities of nanoparticles and vitamins were found to be lowered due to the film’s multicomponent interlayering of peaks. These structural features demonstrate that the composite matrix partly conceals the nanoparticles' crystallinity^21^, resulting in the complex development and successful cross-linking of film components to efficiently function as wound dressings.

### Physical characterization of films

The solvent-casting approach was adopted to fabricate WG films as (i) referenced films, (ii) by adding NPs, and (iii) NPs-vitamin composite (WG/P/Zn/A, WG/P/Zn/E, WG/A/Ag/A, and WG/A/Ag/E). The incorporation of NPs and nanocomposite into WG films changed the physical appearance of the film from translucid to semi-transparent and opaque (Fig. 4i–l). Reduction in transparency caused by the addition of NPs to the film structure has been documented by several reports^52^. Physical appearance, weight variance, thickness, and degree of folding flexibility are all important factors for investigating the potential role of films as a bioactive drug delivery carrier (Table 2). The film thickness and weight are crucial because these affect the structural and functional aspects of the films. Glycerol as a plasticizer improved the integrity of the WG film structure. Among the WG films, vitamin-loaded and unloaded films were robust and flexible, easy to handle, and could be cut into the requisite shape for use on the targeted wound site.

**Table 2:** Physical appearance, thickness, weight variance, and folding flexibility of WG films (reference) nanocomposite WG films and NPs-vitamin loaded WG films.

WG-film formulations	Physical appearance	Weight (g)	Thickness (µm)	Folding flexibility
WG/P	Translucid	0.7 ± 0.2^d^	106.6 ± 0.1^e^	25 ± 5^d^
WG/A	Translucid	0.8 ± 0.2^c^	80.5 ± 1.34^f^	32 ± 3^b,c^
WG/P/Zn	Semi-transparent	0.85 ± 0.13^c^	185.3 ± 0.73^b^	29 ± 2^c^
WG/A/Ag	Opaque	1.3 ± 0.08^b,c^	135.6 ± 0.5^d^	35 ± 4^b^
WG/P/Zn/A	Semi-transparent	1.41 ± 0.06^a,b^	205 ± 1.7^a,b^	38 ± 2^a^
WG/P/Zn/E	Semi-transparent	1.45 ± 0.07^a^	225 ± 1.4^a^	38 ± 3^a^
WG/A/Ag/A	Opaque	1.38 ± 0.07^b^	165 ± 1.6^c^	37 ± 4^a,b^
WG/A/Ag/E	Opaque	1.42 ± 0.08^a,b^	205 ± 1.8^a,b^	39 ± 4^a^

The different letters within the column mean the statistical difference between treatments by LSD test p ≤ 0.05.

### Swelling response of WG films

**Figure 5:**
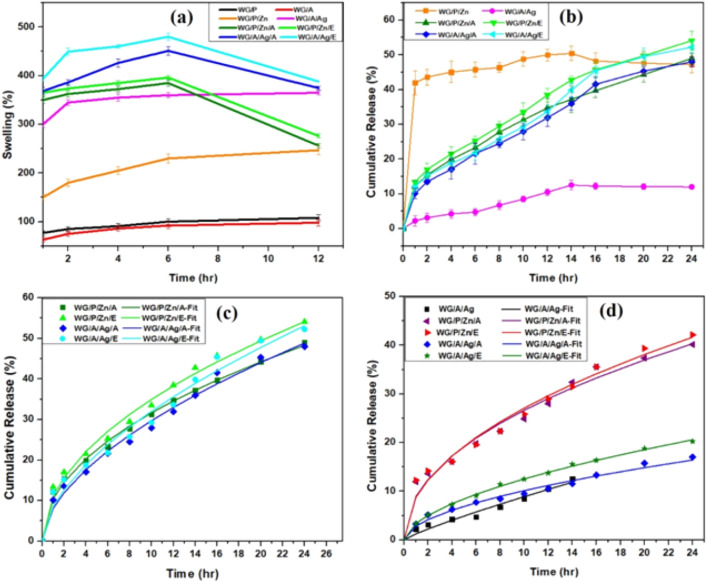
(**a**) Swelling response of WG films (**b**) In-vitro Ag NPs, ZnNPs from unloaded WG/P/Zn and WG/A/Ag films**,** and VA, and VE release from vitamin-loaded nanocomposite WG films. Bars show standard derivations of three different means (mean and SD); (**c**) In-vitro VA and VE release data fitted to Korsmeyer and Peppas drug diffusion model (**d**) In-vitro ZnO NPs and Ag NPs release data fitted to Korsmeyer and Peppas model. Symbols represent the experimental data, Solid lines represent the predicted model.

The swelling response and structural integrity of wound healing films are critical for their clinical applications in skin tissue remodeling^1^. The swelling capacity of wheat gluten composite films, either vitamin-loaded or unloaded, reflects the films ability to absorb fluid, which has a significant impact on the diffusion of the contained bioactive substances. Swelling of the films was observed continuously for 12 h at pH 7. Optimum swelling capacity was achieved within a few hours and the films preserved structural integrity during immersion time. During first 6 h of immersion, the swelling of the films followed Fick's law, demonstrating that the sorption of water molecules is controlled by diffusion. NPs-vitamin composite loaded WG films had a higher swelling index during 6 h than other films while control films showed a lower but progressive swelling response throughout the time period (Fig. 5a). The WG/P and WG/A as WG reference films showed the lowest levels of liquid absorption i.e. 108% and 98% respectively. In pH 7 medium, WG/P/Zn and WG/A/Ag films showed the maximum liquid absorption pattern with 247% and 365%, respectively after 12 h.

WG/P/Zn/A and WG/P/Zn/E films had improved liquid absorption by a maximum swelling index of 365% and 396% in the first 6 h, respectively, which gradually decreased by 256% and 275%, respectively in lateral hours. Similar findings were observed for WG/A/Ag/A (451%) and WG/A/Ag/E films (480.05%) during the first 6 h (Fig. 5a). The presence of vitamins considerably reduced the swelling response of the nanocomposite films, regardless of the type of vitamin used. The decrease in swelling of films reflects the hydrophobic behavior of vitamins A and E. However, when compared to unloaded nanocomposite WG films, there was a significant increase in the swelling capacity of NPs-vitamin loaded WG films i.e. 67% by WG/P/Zn/A and 72% by WG/P/Zn/E films to their respective control film WG/P/Zn. Similarly, the swelling index of vitamin-containing Ag NPs films was also found to be higher than unloaded WG/A/Ag film i.e. 25% by WG/A/Ag/A and 33% by WG/A/Ag/E films.

The interaction of NPs-vitamin composite resulted in the formation of pores/pits in the film network (Fig. 4a–d). Such pits or poresstimulate liquid uptake from the surroundings^53^. However, due to the hydrophobic nature of vitamins, early desorption of water molecules from the film matrix causes a reduction in swelling capacity. Though early swelling is preferable because it increases pore size, which promotes cell adhesion and proliferation in a three-dimensional structure^54^. Prolonged swelling may cause the surrounding tissue to lose mechanical integrity and macerate^1,55^. Therefore, significant fluid absorption by the films is desirable to absorb excess exudate, and maintain the integrity of the applied dressing and surrounding skin tissues^56^. As a result, the liquid absorption values recorded in this study are consistent with those recommended for wound dressing treatments. Many studies have demonstrated that fibroblasts and keratinocytes migrated more rapidly when cultured under neutral environments^57–59^. Some studies report that a slightly acidic pH is favorable for wound healing by controlling the skin infections in wounded skin^60,61^ While, Kruse et al.^59^ revealed that ideal pH for wound contraction and healing is 7.

The cohesive characteristics of wheat gluten and cross-linkers establish strong networks with NPs and vitamins enabling optimal liquid retention capacities, efficiently permeating the antioxidative substances into the system, and ensuring the structural integrity of film materials. Therefore, these could be promising therapeutics for skin tissue re-modeling applications.

### Release study of NPs/ions and vitamins from WG films

The release of NPs/ions and vitamins from films signifies potential interactions between the NPs/ions, vitamins (drug), and the gluten polymer and their impact on the rate and mechanism of drug release. A progressive release pattern of VA and VE from WG/P/Zn/A, WG/P/Zn/E, WG/A/Ag/A, and WG/A/Ag/E films was noted from 1st to 24 h (Fig. 5b**).** The 1^st^ hr release of vitamin A from WG/P/Zn/A and WG/A/Ag/A films was 12% and 10%, followed by continuing progressive release over the next 24 h. After 24 h of immersion, the cumulative VA release from films achieved a plateau of around 49% (for WG/P/Zn/A) and 48% (for WG/A/Ag/A). The initial release of VE from WG/P/Zn/E and WG/A/Ag/E films was 13% and 12% after 1st h, respectively. The cumulative release of VE from WG/P/Zn/E and WG/A/Ag/E films, reached around 54% and 52% after 24 h of immersion. It was found that WG films retained their original structure after 24 h at 37 °C. The accumulation of vitamins near the surface of the films caused the initial rapid outflow of VA and VE into the liquid medium. The majority of the entrapped vitamins were not released into the system. This is believed to be the result of some vitamins being deeply embedded in the film structure where they cannot diffuse due to the compact matrix.

The progressive release of ions/NPs from NPs-vitamin loaded WG films is also presented in Fig. 5d. The maximum Zn^++^/ZnO NPs release was recorded in WG/P/Zn/E films (42%) followed by WG/P/Zn/A films (40%) whereas WG/A/Ag/A and WG/A/Ag/E films released 17% and 20% Ag^+^/NPs, respectively. However, the maximum release of ions/NPs was recorded up to 24 h continuously from VA and VE loaded films. Overall, the WG/P/Zn/E matrix showed the highest release behavior in terms of vitamins and ZnO NPs/ions release than the WG/P/Zn/A matrix whereas the WG/A/Ag/E matrix also released the highest rate of vitamins and Ag NPs/ions than WG/A/Ag/A matrix.

The Korsmeyer and Peppas model was applied to interpret the findings from in vitro release studies of ions/NPs and vitamins. The model suggests that expansion and relaxation of the film matrix is critical in promoting vitamin and ions/NPs release. This indicates that various mechanisms may be responsible for release kinetics^62^. The kinetic modeling of a drug release from its host matrix is frequently described using the Korsmeyer–Peppas equation^63^. Korsmeyer–Peppas “n” value (0.45 < n < 0.89) characterize different release mechanism and the model predicts that vitamins and ions/NPs released from NPs-vitamin loaded WG films is non-Fickian, with an anomalous type diffusional transport mechanism (Fig. 5c,d). The anomalous transport mechanism demonstrates both diffusion and relaxation-controlled release of bioactive substances (vitamins and ions/NPs) from composite films. Generally, films with greater “n” values have higher relaxation response^30,64^. The highest n value was found by Ag/VE film (n = 0.57), followed by Ag/VA (n = 0.56), Zn/VE (n = 0.49), and WG/P/Zn/A (n = 0.48) films. Furthermore, the non-Fickian anomalous diffusion mechanism also contributes in the case of ions/NPs released from NPs-vitamin loaded films (Table 3). The release constant (k) is proportional to the diffusion constant and thus is dependent on the physical and structural characteristics of both the drug and host matrix. The drug release rate is indicated by its k value^65^. The strong agreement between experimental data and the equation confirm the validity of the model. Table 3 summarizes the findings of the analyses (k, n, and R^2^ values) that indicate the goodness of fit. The blending of WG, NPs, and vitamins in films promotes an efficient and scalable approach for regulating the duration and rate of vitamin and ion release in wound treatment. The gradual release of vitamins, NPs, and ions is a fundamental aspect of wound therapy.

**Table 3:** Estimated NPs and vitamins release kinetical parameters obtained from fitting experimental data to the Korsmeyer and Peppas model.

WG Films	Release	K	n	R^2^	Release mechanism
WG/P/Zn/A	VA	10.40207	0.483139	0.99843	Anomalous transport
	ZnO NPs/ions	8.959412	0.473346	0.991587	Anomalous transport
WG/P/Zn/E	VE	11.06709	0.499578	0.996451	Anomalous transport
	ZnO NPs/ions	8.732987	0.491852	0.991428	Anomalous transport
WG/A/Ag/A	VA	8.022721	0.568618	0.995	Anomalous transport
	Ag NPs/ions	2.839913	0.552313	0.99334	Anomalous transport
WG/A/Ag/E	VE	8.472682	0.57761	0.99016	Anomalous transport
	Ag NPs/ions	3.402299	0.566485	0.999248	Anomalous transport
WG/A/Ag	Ag NPs/ions	1.239565	0.855526	0.986511	Anomalous transport

On the other side, the model also successfully represents anomalous diffusion-type behavior, in the case of Ag NPs/ions release from WG/A/Ag film (Fig. 5d), a non-Fickian diffusion (case II) driven process (n = 0.85). For WG/P/Zn unloaded films, an initial quick release of Zn^++^/ZnNPs was observed, followed by a progressive release pattern from 1 to 14 h, indicating the hindered Fickian diffusion to Fickian diffusion type release mechanism (Fig. 5b). However, due to the initial burst release of the NPs from the WG/P/Zn films, only the release kinetics of the Ag^+^/NPs from the WG/A/Ag were analyzed using the Korsmeyer-Peppas equation. The in vitro release of Ag NPs and ZnO NPs (within 1–14 h) over time in the buffer media is kinetically regulated dissolving pattern, governed primarily by Fickian and non-Fickian diffusion mechanisms^18^. As a result, it is expected that drug release from all the WG films will be driven by the diffusion process. Unagolla and Jayasuriya^65^, Aranates et al.^30^ and others demonstrated comparable drug release kinetics for antibiotics and nanoparticles^23,66^. WG films released fewer ions/NPs than the total count of NPs found in the WG polymer network. It is also essential to avoid the systemic toxicity of NPs for safety reasons. Conclusively, it is suggested that gluten matrix facilitates a higher release of vitamins than NPs. Moreover, the NPs in the gluten matrix have redox properties that protect VA and VE from oxidation, leading to less degradation during the release process.

### Antioxidant potential

The addition of antioxidant agents in wound dressing improves the wound healing process by limiting ROS generation. The antioxidant potential of NPs, NPs-vitamin loaded WG films, and unloaded nanocomposite WG films was assessed using DPPH and ABTS radical scavenging assays. NPs-vitamin composite loaded WG films had higher radical scavenging activity than control films (Fig. 5a). The highest percentage of radical scavenging (DPPH) 90% and 89% was demonstrated by WG/P/Zn/E and WG/A/Ag/E films, respectively. VE-loaded WG films also showed significant ABTS scavenging activity; 46.06% by WG/P/Zn/E and 44.2% by WG/A/Ag/E. WG/A/Zn/A film also showed a significant percentage scavenging with 88% (DPPH) and 42.64% (ABTS), followed by WG/A/Ag/A film, which had an overall scavenging potential of 86% (DPPH) and 40.43% (ABTS). These findings suggest that the film's surface contained a range of bioactive components, such as NPs, VA, and VE, which function synergistically and enhance the antioxidant potential of NPs-vitamin loaded WG films. Vitamin E is recognized for its high antioxidant potential due to the presence of the OH group on its chromanol ring structure^31^. Vitamin E prevents ROS attack on cell membranes and lipids by causing the upregulation of several signal transduction pathways^67^. The skin membrane is shielded from free radical damage by the antioxidative properties of vitamin E^1^. VA also prevents skin lipid peroxidation by exfoliation, which speeds up cell repair^25,31^. Furthermore, the property of ZnO NPs and Ag NPs to contribute electrons or hydrogen ions to mitigate the oxidative stress (caused by unstable free radicals) in the reaction system is a possible explanation for their potential to scavenge DPPH and ABTS radicals. According to Jabirili et al.^48^, the addition of ZnO to the gluten matrix increased the radical scavenging capacity of films.

Production of reactive oxygen species (ROS) is one of the defense strategies that inflammatory cells use to fight invading pathogens. High ROS levels at the wound bed can cause serious tissue injury as well as neoplasm formation, which may mimic the rate of healing by damaging cellular membranes, lipids, DNA, and proteins. ROS negative effects can be minimized by delivering "antioxidants" to the injured site^68^. Antioxidants "donate" themselves by neutralizing free radicals, which causes them to be less destructive^22,68^. This mechanism allows antioxidants to speed up and accelerate the healing process. Thus the addition of vitamins A and E to WG nanocomposite film can improve the product with excellent antioxidant potential.

### Antibacterial potential

An imbalance between localized pathogenic factors and the stability of immune defenses at the wound site hinders wound healing and promotes the invasion of pathogenic bacteria. *P. aeruginosa, E. coli, S. aureus, and S. epidermidis* are the most common bacterial strains found in injured skin areas^22^. Disc diffusion and MBD assays were followed to investigate the antibacterial activity of fabricated films against *P. aeruginosa, E. coli, K. pneumoniae, S. aureus, MRSA,* and* S. epidermidis.*

The incorporation of vitamins into the nanocomposite WG films improved their antibacterial potency against tested bacterial strains (Table 4). The vitamin-loaded and unloaded WG/P/Zn films significantly inhibited bacterial strains except MRSA. The maximum inhibition (12 mm) was seen by WG/P/Zn and WG/P/Zn/E against *P. aeruginosa, and K. pneumoniae,* respectively while WG/P/Zn/E was also found active against *S. epidermidis.* The inhibitory zones of vitamin ZnO NPs composite containing films may be due to the higher release rate of Zn^++^ or vitamins from the film surface. ZnO NPs/Zn^++^ release stimulates the generation of reactive oxygen species (ROS) on the bacterial cell membrane, resulting in cellular constituents leakage and, eventually, cell death^20^. Vitamin-loaded Ag NPs films were also found potent for bacterial resistance than the unloaded WG/A/Ag films. WG/A/Ag/E film had the strongest antibacterial activity against *S. epidermidis* (13 mm), *K. pneumoniae* (12 mm), and *E. coli* (12 mm) due to their NPs and VE molecules that release from the films and penetrate inside the bacterial cell causing damage to bacterial machinery. However, significant percent inhibition was also shown by ZnO NPs against *S. aureus* and Ag NPs against *E. coli* and *S. aureus* with 12 mm of the inhibitory zone. MRSA was observed to be the most resistant bacteria against all film samples because all of the incorporated vitamins and NPs were unable to contact directly with the bacteria growing on the agar plates in the disc diffusion approach^69^.

**Table 4:** Antibacterial potential of ZnO NPs, Ag NPs, NPs-vitamin loaded and unloaded nanocomposite WG films.

WG film formulations	Antibacterial potential (ZOI; mm)					
	*E. coli*	*P. aeruginosa*	*K. pneumoniae*	*S. aureus*	*MRSA*	*S. epidermidis*
WG/P/Zn	11 ± 0.5^a,b^	12 ± 0.6^a,b^	10 ± 1.5^b^	12 ± 0.5^a^	8 ± 0.8^a,b^	9.5 ± 0.7^c^
WG/A/Ag	10 ± 0.9^b^	11 ± 0.5^b^	9 ± 0.7^b,c^	7 ± 1.4^bc^	7.5 ± 0.4^b^	9.5 ± 1^c^
WG/P/Zn/A	10 ± 1.6^b^	10 ± 1.5^c^	10 ± 1.6^b^	12 ± 0.5^a^	9.5 ± 1^a^	10 ± 0.6^b,c^
WG/P/Zn/E	11 ± 0.6^a,b^	12 ± 0.7^a,b^	12 ± 0.5^a^	10 ± 1^b^	9 ± 1.3^a^	12 ± 0.5^a,b^
WG/A/Ag/A	11 ± 1^a,b^	10 ± 2.5^c^	11 ± 0.7^a,b^	11 ± 0.6^a,b^	8.5 ± 0.5^a,b^	10 ± 1.5^b,c^
WG/A/Ag/E	12 ± 0.5^a^	11.5 ± 0.8^b^	12 ± 0.8^a^	9.5 ± 1.5^b^	9 ± 0.5^a^	13 ± 0.8^a^
ZnO NPs	8 ± 2^c^	10 ± 0.7^c^	8 ± 1^c^	12 ± 0.5^a^	9 ± 0.35^a^	11 ± 1.4^b^
Ag NPs	12 ± 0.5^a^	13 ± 0.5^a^	10 ± 0.6^b^	12 ± 0.75^a^	9 ± 1.5^a^	10 ± 0.5^bc^

Cefixime and Roxithromycin were used as positive control in the antibacterial assay. Cefixime showed 20 ± 0.5 mm ZOI against *P. aeruginosa*, 15 ± 1 mm against *E. coli*, 19 ± 1 against *K. pneumoniae*, and *Roxithromycin* showed 20 ± 1 mm ZOI against *S. aureus*, 18 ± 1 mm ZOI against *MRSA*, and 18 ± 1 mm ZOI against *S. epidermidis*. The different alphabetical letters within the column mean the statistical difference between treatments by LSD test p ≤ 0.05.

**Figure 6:**
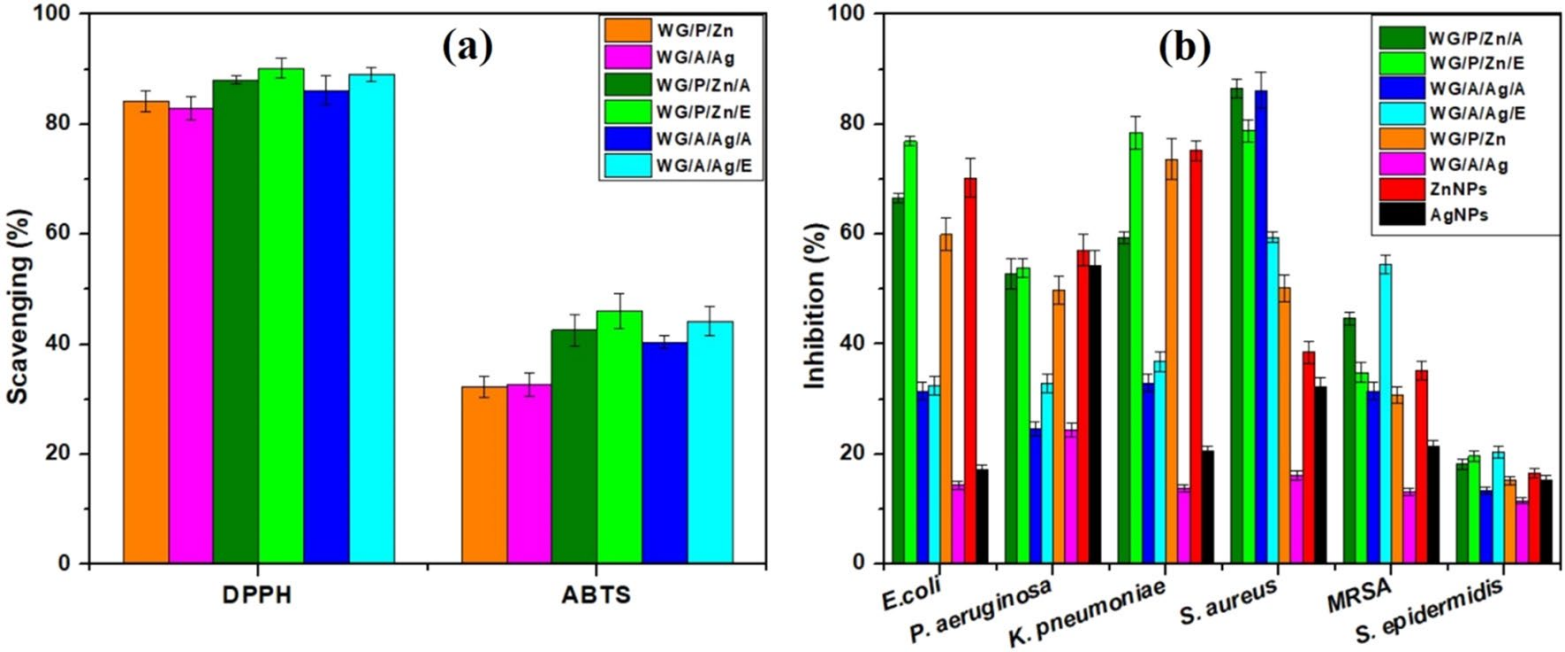
(**a**) Free radical scavenging potential (%) of WG films, (**b**) Bacterial growth inhibition (%) of WG films.

By employing MBD approach, the films responded better to bacterial inhibition in comparison to the disc diffusion approach (Fig. 6b). WG/A/Ag/E and WG/P/Zn/A films efficiently inhibited the growth of MRSA by 54% and 44%, respectively. WG/A/Ag/E and WG/P/Zn/E films were effective against tested Gram-positive and Gram-negative bacterial strains except *S. aureus*. However, 86% reduction of *S. aureus* was observed by WG/A/Ag/A and WG/P/Zn/A films whereas the minimum reduction in *S. epidermidis* cell count was noted by all film samples (Fig. 6b). The presence of a peptidoglycan surface layer of *S. epidermidis* (Gram-positive bacteria) may act as a barrier that prevents NPs and vitamin molecules from penetrating and, as a result, low activity was observed.

The micro broth method demonstrated that the unloaded nanocomposite films (WG/P/Zn, WG/A/Ag) had a substantial capacity to lower the bacterial load. ZnO NPs were more resistant to all strains than Ag NPs. The released ZnO NPs or Zn^++^ are closely linked to bactericidal actions^70^. Likewise, the relatively few Ag^+^ released from WG/A/Ag film justified its mild bacterial inhibition and low toxicity. The difference in antibacterial potential of film is attributed to differences in the structure and chemical composition of the bacterial cell membranes^71^. However, NPs-vitamin loaded WG films showed higher antibacterial potential than their respective control films. Metallic NPs have been widely considered as alternative solutions to antibiotics due to potency and affordability. These nanoparticles have been shown to improve the wound healing process by reducing localized skin inflammation, and keeping pathogens away from the skin^72^. Gram-positive bacteria may be inhibited by disrupting bacterial cell membranes, inhibiting DNA replication, RNA synthesis, and protein synthesis, while Gram-negative candidates may be killed by altering barrier characteristics and permeability of the membrane^73^.

### In vivo wound healing potential

#### Wound contraction analysis

**Figure 7:**
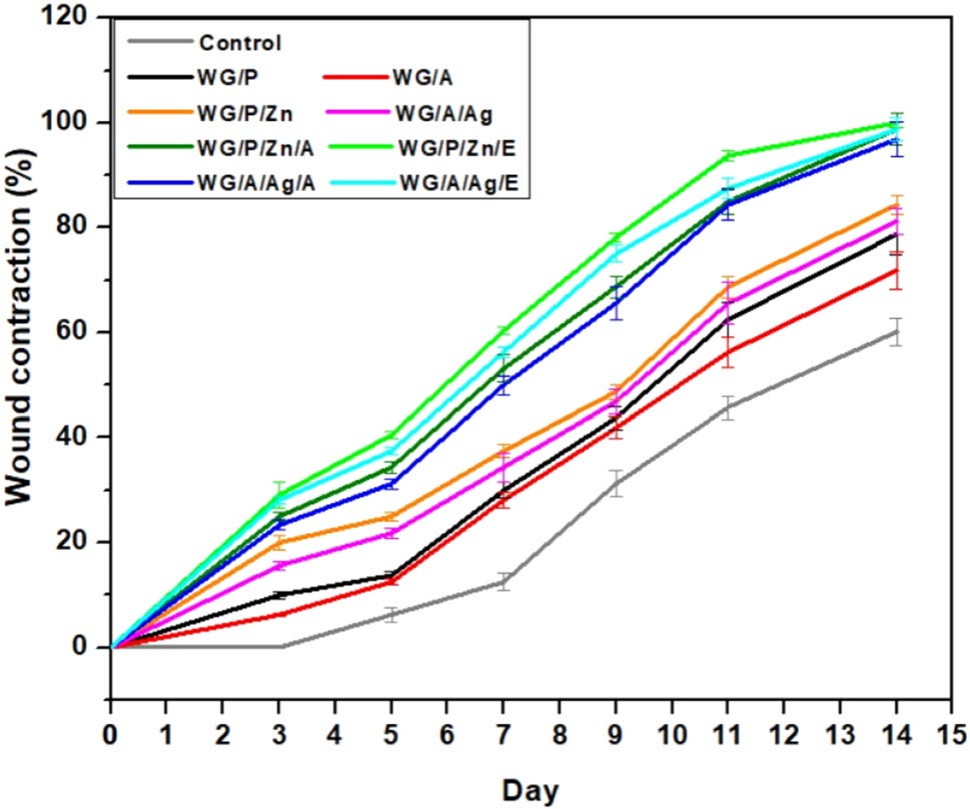
Wound contraction (%) in mice by WG films on 3rd, 5th, 7th, 9th, 11th, and 14th day.

The wound healing potential of designed films as wound dressingswas studied in vivo on burn-injured mice models. The NPs-vitamin loaded WG dressings showed remarkable wound contraction potential (Fig. 7). Repeated application of NPs-vitamin loaded dressings greatly accelerated the healing process, resulting in speedy wound contraction, earlier scab removal, and reduced scarring (Fig. 7). In the WG/P, WG/A, and control group (untreated), both inflammatory and infectious states were observed, implying that they were not intended to cure the wound. However, when Ag NPs and ZnNPs were incorporated into the WG films, wound healing efficiency was considerably satisfactory. However by the addition of VA and VE to the nanocomposite WG films, wounds healing significantly improved. Application of WG/P/Zn/E, WG/A/Ag/E, WG/P/Zn/A, and WG/A/Ag/A films showed no signs of inflammation or infection and effectively healed wounds.

**Figure 8:**
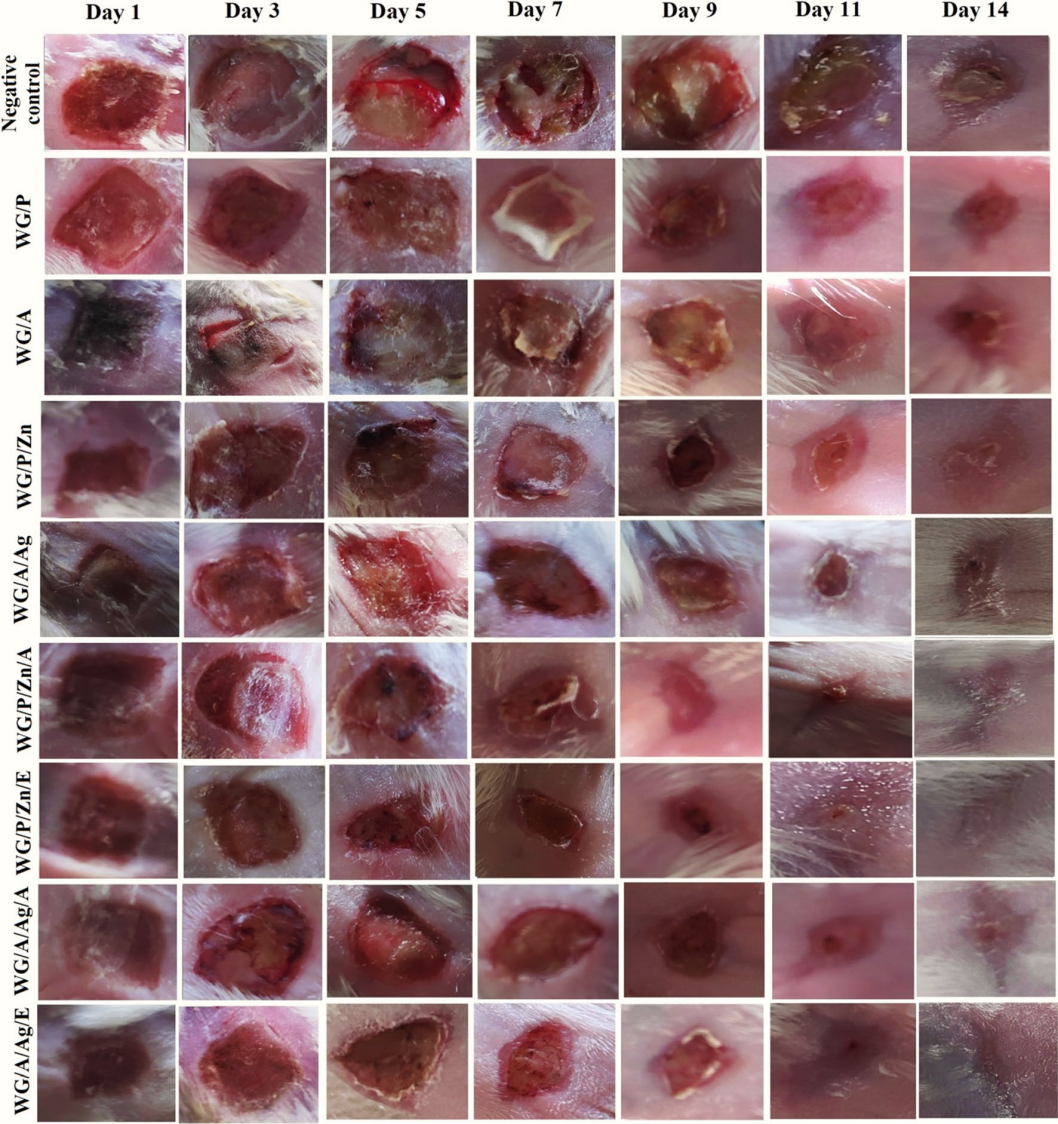
Burn wound contraction of mice skin on different days (horizontal) and in vivo wound healing efficiency of WG films (vertical).

The relative wound areas estimated at various stages following the first injury are shown in Fig. 8. According to the results, on the 3rd day, no wound contraction (%) was noted for the control group, and minimal wound contraction for WG/P (10%), WG/A (6.25%) groups. WG/P/Zn, WG/A/Ag, WG/P/Zn/A, WG/P/Zn/E, WG/A/Ag/A, and WG/A/Ag/E groups wound contraction was found to be 20%, 15.62%, 25%, 29.12%, 23.37%, and 28.12%, respectively. All the mice exposed to NPs-vitamin loaded dressings were found to be physically normal. The wound was adherent to the film because the exudate was significantly absorbed by the NPs-vitamin loaded dressing. The wound got infected in the mice of negative control, WG/A, and WG/P groups, resulting in the edematous area with ulceration and slower wound contraction. The healing process continued and on the seventh day, NPs-vitamin WG films treated groups had a considerable reduction in the wound area, with a maximal contraction of around ≥ 50% (Fig. 8). The antibacterial properties of the ZnO NPs, Ag NPs, and vitamins in the dressing kept the wound area uninfected. In contrast, persistent wound infection and inflammation were found in the mice of the control, WG/A, and WG/P groups. On the 9th and 11th day, NPs-vitamin loaded groups healed faster with maximum wound contraction (Fig. 8). However, mice in the control and WG/A groups showed minimal while WG/P groups exhibited moderate healing behavior and wound contraction (Fig. 8). Consequently, on the 14^th^ day, the percentage of wound closure for the control group was 60.12%, 78.75% (WG/P), 71.87% (WG/A), 84.37% (WG/P/Zn), 81.25% (WG/P/Ag), 98.75% (WG/P/Zn/A), 100% (WG/P/Zn/E), 96.87% (WG/A/Ag/A), and 98.75% (WG/A/Ag/E). According to Kyriakides et al. thrombospondin-2 (TSP-2) expression level upregulates on the 7th day after wounding and have been linked to re-epithelialization and faster wound closure in mice^74,75^. Fast and significant wound contraction by nanocomposite films could be due to biocompatibility with injured tissue and better efficiency of the vitamins associated OH group for healing potential. Scabs or scars were not found in NPs-vitamin loaded dressings except in the WG/A/Ag/A group where minimal scarring was observed. The NPs-vitamin loaded wound dressings stimulated wound contraction, re-epithelialization, as well as tissue thickness by increasing collagen deposits, migration of certain skin and immune cells, and angiogenesis. Such outcomes are attributable to the complex film structure, liquid holding capacity, release of bioactive agents, and significant antibacterial potential of NPs-vitamin loaded dressings. Vitamin-rich dressing may enhance keratinocyte and fibroblast proliferation, boost collagen reserve regeneration, reduce inflammation, and speed up wound healing^1,23,30,31,76^. Prior studies have also shown that chitosan-Ag-ZnO composite dressings can promote up to 92% wound closure after six days of healing^77^. Sethuram et al.^18^ also demonstrated that PVA-Ag NPs have a vital role in re-epithelization and a speedy skin regeneration process in Wistar albino rats. PVA and PVP function as basic polymers to generate complex film formation networks, promote the uniform dissolution of the constituents, and enhance their bioabsorbility^18,78^. The increased exude absorption and the release of NPs and vitamins into the wound micro-environment could also be possible through the contraction and relaxation of the polymeric chains^77^.

#### Histopathological analysis

**Figure 9:**
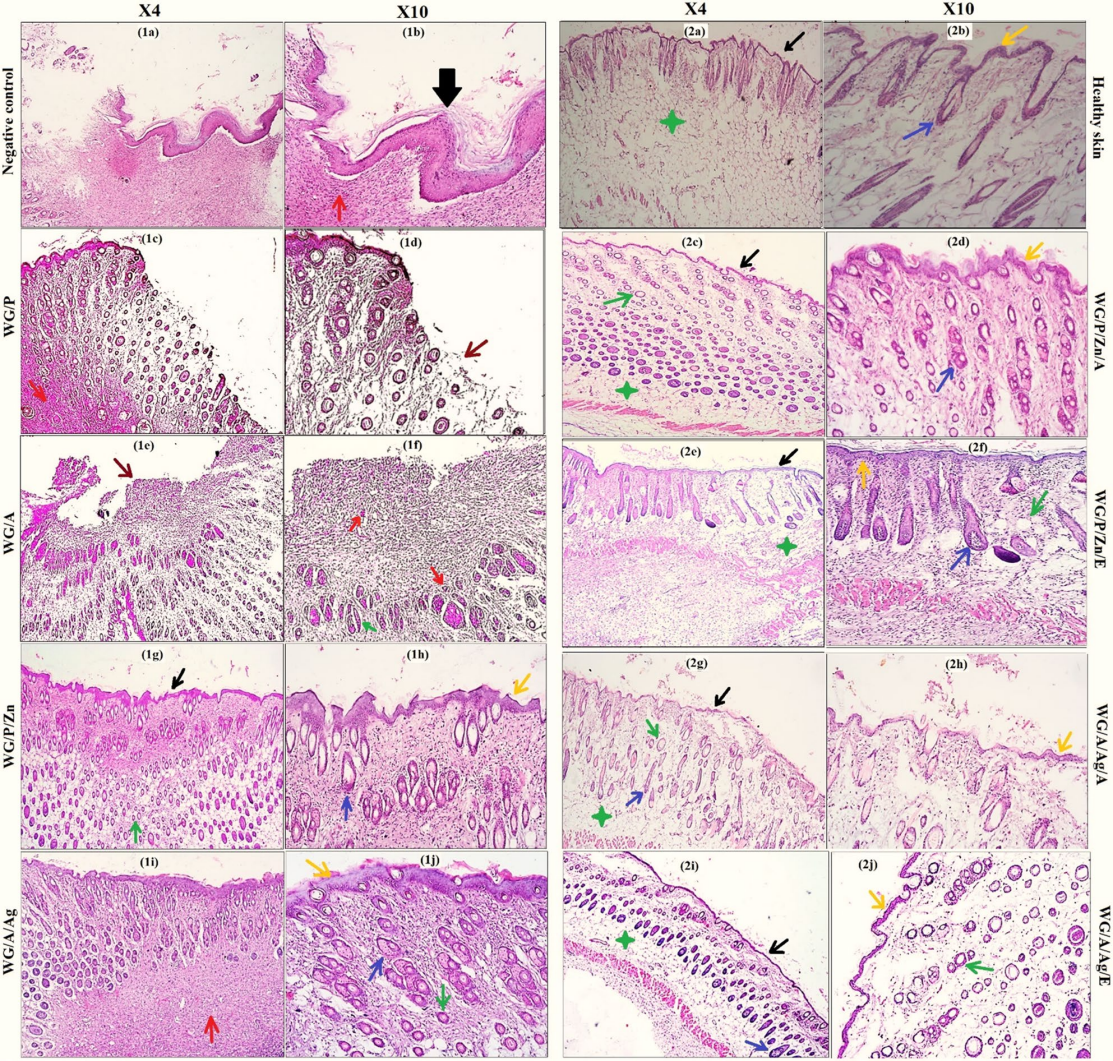
Histological sections analysis of the excised skin of mice at 14 days of post wounding treatment. Highlighted figures represent arrows for: thick epidermis (thick black arrow), thin epidermal layer (thin black arrow), keratinocytes (yellow arrow), collagen deposition with fibroblasts regeneration (green arrow), hair follicles (blue arrow), inflammation (red arrows), detached epithelium/no epidermis (brown arrow), and star indicate adipocytes.

The focus of burn wound treatment remains the selection of compatible biocomposites to accelerate speedy tissue regeneration. After 14 days of burn, NPs-vitamins loaded WG films enhanced the rapid skin restoration process, which involved fibroblast regeneration, collagen resynthesis, hair follicles development, vascularization, and re-epithelization in the burned skin cells. Histological sections of healthy mice skin revealed normal cell distribution in the various layers of the skin, with no signs of inflammatory cells or irregular epidermal layer (Fig. 9,2a,2b). In the negative control group, the histopathological study of the wound after 14 days showed an uneven, disrupted, and thicker epithelium progression (black thick arrow). The wound tissue was filled with debris and accumulated inflammatory cytokines (red arrow) with minimal hair follicle growth (Fig. 9,1a,1b). Minimal to moderate re-epithelialization was observed in the wound treated with WG/Aand WG/P, particularly WG/A where the wound was covered by a medium-thick layer of poorly organized granulation tissue with pericellular edema. WG/P treated group showed proliferation of the epidermal layer with milder inflammation (Fig. 9,1c red arrow), however, detachment of epithelium was observed in some areas (Fig. 9,1c brown arrow). Several micro-environmental stimuli including wound exudate, infection, hypoxia, poor circulation, and increased concentration of TNF-α produced by neutrophils may cause a persistent inflammatory phase within the wounded tissue. All of these factors negatively affect the rate of the healing process, ultimately preventing the wounds to shift from a pro-inflammatory phase^79–81^.

WG/A/Ag dressings demonstrated cellular proliferation of fibroblast, revascularization, and granulation tissues with minimal signs of inflammation (Fig. 9,1i red arrow) and the new generation of hair follicles. WG/P/Zn performed better than WG/A/Ag films in terms of re-epithelialization, fibroblast multiplication, and collagen synthesis. Zn as a micronutrient plays several essential functions in wound healing, such as antimicrobial and anti-inflammation activities, phagocytosis, vascularization, re-epithelialization, and remodeling of skin^82^. In NPs-vitamin loaded WG films groups, no wound damage (scabs) was observed after 14 days of post-wound treatment. There was no evidence of edema or inflammation. Wounds repaired with NPs-vitamin loaded WG films exhibited more stratified epidermal layer than unloaded films, showing that vitamins A and E efficiently enhanced re-epithelialization by stimulating keratinocyte proliferation (yellow arrow). Throughout the healing process, mice skin in these groups showed the maximal reproduction of fibroblast and collagen and was substantially more functional and angiogenic than other groups (Fig. 9). As the remodeling process progresses, granulation tissue formation and re-epithelialization were nearly complete in all groups treated with vitamin-loaded dressings (thin black arrow), however, in WG/A/Ag/A treated group, re-epithelialization was under process (Fig. 9,2g,2h). WG/P/Zn/E treated mice skin had more resemblance to healthy mice skin tissue, with hair follicles in the anagen phase (blue arrow) in comparison to other treatment groups (Fig. 9,2e,2f). WG/P/Zn/A, WG/A/Ag/E, and WG/A/Ag/A dressing also showed maximum fibroblast and collagen synthesis (green arrow), and the early phase of the hair follicle (hair bulb) was observed with no sign of inflammation. It can be related to VE and VA anti-oxidative properties. These vitamins have a role in a variety of biological processes including defending cells and tissues from oxidative stress^1,83^. Vitamins A and E promote keratinocytes and collagen deposition in the repair zone of a wound and aid in wound healing^23,25^. Wheat gluten matrix supports the sustained release system, can facilitate the gradual release of vitamins and NPs in the skin, and significantly minimizes the toxicity risk of NPs. It has also been reported that ZnO NPs and Ag NPs regulate the respiratory signaling pathways of cells so that skin cell regeneration may occur, resulting in proliferation within the requisite duration and hence restoration of the skin surface^28,84^. Ag-based biomedical formulations have the potential to be effective in the treatment of bacterial infections and wound healing^18^. Collagen is the primary structural protein that forms connective tissue and is present mostly in the skin. It is therefore very important to examine the collagen remodeling in the healed skin. As shown in Table 5, the mice groups treated with NPs-vitamin loaded WG films (WG/P/Zn/A, WG/P/Zn/E, WG/A/Ag/E, and WG/A/Ag/A) maximum collagen deposition was observed, reflecting an increase in collagenase activity due to the synergistic interaction of vitamins (A & E), Zn and Ag NPs.

**Table 5:** In vivo physical parameters analysis and histopathological analysis of treated mice from NPs-vitamin-loaded and unloaded WG films, reference WG films and negative control groups.

Treatment groups	Mice physical analysis at day 14				Histological analysis of mice skin samples (day 14)
	Active	Swelling	Redness	Infection	
WG/P	++	+	+	+	Detached epithelial tissues, presence of collagen and fibroblasts, minimal infection
WG/A	++	+	+	+	Poorly organized granulation tissue with inflammation, minimal epithelial tissue regeneration, fibroblast and collagen
WG/P/Zn	+++	−	−	−	Thin epidermal layer, hair follicles, satisfactory synthesis of collagen fibers and fibroblasts, and minimal infection
WG/A/Ag	+++	−	−	−	Moderate epidermal layer, hair follicles, minimal infection, and re-synthesis of collagen fibers and fibroblasts
WG/P/Zn/A	+++	−	−	−	well-defined thin epidermal layer, hair follicles, maximum collagen and fibroblasts, adipocytes, and no infection
WG/P/Zn/E	+++	−	−	−	well-defined thin epidermal layer, hair follicles at anagen phase, maximum collagen and fibroblasts, adipocytes, and no infection
WG/A/Ag/A	+++	−	−	−	Thin epidermal layer with moderate keratinocytes, hair follicles, maximum collagen and fibroblasts, adipocytes, and no infection
WG/A/Ag/E	+++	−	−	−	well-defined thin epidermal layer, hair follicles, maximum collagen and fibroblasts, adipocytes, and no infection
Negative control (untreated)	+	++	++	++	Uneven, disrupted, and much thicker epithelium, dense inflammation, no fibroblasts

Mean values from 6 mice. (+++ for maximum, ++ for moderate, + for mild, and − for absence).

Li et al.^23^ reported significant healing effects of gelatin formulations loaded with vitamins A & E while treating a burn wound. Arantes and his colleagues also noted comparable results including decreased inflammation and scarring with enhanced collagen deposition by using SLN-ATRA composite in chitosan films to treat diabetic wounds^30^. Numerous studies have also proved that VE can restore damaged skin caused by UV exposure. This vitamin offers the potential to reduce numerous pathological conditions in the skin because its role is vital to the body's capacity to fight against free radicals^85,86^. Vitamin A also promotes the immune system, and angiogenesis, and restores collagen production in skin wounds^87^.

## Conclusion

In this study, the interaction between wheat gluten and NPs was studied to evaluate the functionality of wheat gluten nanocomposite in the film. Wheat gluten nanocomposite showed remarkable biological and physical properties and can be an appropriate choice for dressing preparation. Further, vitamin A and vitamin E were successfully coated onto the ZnO and Ag NPs, and WG films loaded with NPs-vitamin composite were successfully casted. FTIR confirmed the coating of vitamins to NPs and the integration of NPs-vitamin composites into WG film. The addition of vitamins into WG film altered the film surface morphology and demonstrated bumpy structure in vitamin E-based WG films and dense network-like structure in vitamin A-based WG films. Incorporation of NPs-vitamin composites into WG films had a substantial impact on physical characteristics and hydrophilicity. Furthermore, the progressive release of the VE, VA, and NPs/ions was observed for 24 h. The presence of ZnO and Ag NPs, and even vitamins (A and E) in the WG films makes them effective against Gram-positive and Gram-negative bacterial strains and have antioxidative potential. Based on in vivo testing on burn-injured mice, the designed NPs-vitamin loaded WG films showed faster wound reconstruction, and histological observations (re-epithelization, fibroblast regeneration, collagen resynthesis, adipocytes, and hair follicle development) suggest their applicability as an effective wound dressing product. Its beneficial application in clinical practice may provide a viable treatment route for achieving scar-free wound care.

## Data Availability

The data and materials have been reported in the manuscript.
